# Effects of stress during commercial hatching on growth, egg production and feather pecking in laying hens

**DOI:** 10.1371/journal.pone.0262307

**Published:** 2022-01-04

**Authors:** Louise Hedlund, Per Jensen

**Affiliations:** IFM Biology, Linköping University, Linköping, Sweden; University of Illinois, UNITED STATES

## Abstract

Every year, billions of egg layer chicks around the world are hatched under highly stressful, industrial circumstances. Here, it is investigated how the stressful procedure in the commercial hatchery, including incubation, hatching, processing, and transport affects the chicks with regards to traits relevant for the egg production industry. These traits were compared to those of a control group hatched in a small incubator and handled gently och quietly in a quiet room without any processing and transport. The chicks were weighed at hatch and at eight additional time points: 4 days, 1 week (w), 2 w, 3 w, 5 w, 8 w, 20 w and 25 w of age. Feather pecking was studied at 15 w of age and damages to the feathers and injuries on the comb and wattle were assessed at 25 w of age. From 19 w of age, eggs were collected on three days per week, counted and weighed. Chicks from a commercial hatchery had a lower hatch weight than control chicks (p<0.001). At 20 w of age, the weight of the commercial hatched chicks was still numerically lower, although this did not reach statistical significance. Commercially hatched chicks tended to show more feather pecking behaviour at 15 w of age compared to control chicks (p<0.1), although feather condition at 25 w of age showed the opposite pattern. Regarding production, commercially hatched chickens laid fewer (p<0.05) and smaller (p<0.05) eggs than chicks hatched and handled under calm circumstances. From this experiment, it is concluded that the stressful experience in the commercial hatchery has an overall negative effect on traits relevant for the industry.

## Introduction

Every year, billions of egg layer chicks around the world are hatched under industrial circumstances [1]. The hatchery procedure is highly standardized and does not differ considerably between countries [2]. Briefly, upon arrival to the hatchery, the eggs are inserted in large scale incubators (each containing tens of thousands of eggs) and are incubated in darkness with highly controlled temperature and humidity. The noise level in the incubators is about 90 dB (caused mainly by fans) and during the last 3 days of incubation, the chicks are often exposed to formalin fumigation. Chicks hatch on average at day 21 of incubation but are routinely kept in the incubator another day to allow for late hatchers.

When hatched, the racks with chicks are tilted on a conveyer belt system where the birds are sex sorted, vaccinated, beak trimmed (in most countries; however, not in Sweden), and packed in transport boxes. Our research group has previously shown that the treatment in the commercial hatchery is stressful for the animals [3]. It affects their corticosterone (CORT) levels, weight, behaviour and cognitive judgement bias [2–4].

Research has shown that stress early in life affects chickens not only with respect to behaviour, but also in traits relevant for production. For example, several stressors seem to affect growth and weight in chickens. Broiler chicks [5–7] as well as layers [8] react to early temperature stress with reduced weight gain. Early feed restriction affects body weight in broiler chickens [9–11], White Leghorn egg layers [8], quails [12, 13] and turkeys [9], even if chickens seem to catch up and show compensatory growth in just a few weeks [9, 11, 14]. Also, stress pre-hatch can affect the chicks’ body weight and growth, caused by reduced gas exchange [15] and in-ovo exposure to CORT [16].

One trait with high relevance for welfare as well as for production is feather pecking (FP), which is the behaviour of pecking and pulling out feathers of other birds and can vary from gentle to severe. Gentle FP is more of an explorative behaviour whereas severe FP can cause damage to feathers and skin of the recipient. These are two separate behaviours where gentle FP does not seem to necessarily develop to severe [17]. Whereas gentle FP rarely impact the birds negatively, severe FP in laying hens is a welfare problem as it leads to injuries and sometimes cannibalism and mortality. It is also an economic issue because of decreased weight as well as increased mortality and food consumption in defeathered birds [17–19]. Therefore, identifying factors that are of relevance for the development of severe FP is of interest for the industry but also for animal welfare.

Several factors in the chicks’ early-life history have been shown to affect the development of FP. It is most often considered to be a redirected behaviour from dust bathing and ground pecking, where access to sand, straw or grain during the rearing period seem to reduce FP later in life [18, 20–22]. It can also be regarded as part of the normal behavior repertoire and a form of social exploration [23]. Research has shown that there are lower mortality levels due to cannibalism and FP when the chicks are allowed to socialize with the hen during the first weeks of life [24]. Is it also known that chicks reared under dark brooders perform significantly less FP than chicks reared under a regular heatlamp [25]. Pre-hatch light exposure during incubation has also been shown to increase the level of FP in chickens [26, 27].

The most obvious trait that is relevant for the industry is number of eggs laid as well as egg weight and quality. From previous research, it is known that stress can reduce egg quality and egg weight in chickens [8, 28, 29]. However, how early-life stress associated with commercial hatching affects chickens’ production later in life, is to our knowledge not yet investigated.

In this study, it was investigated if weight development, feather pecking and egg production are affected by early stress in commercial hatching. The hypothesis was that chicks from a commercial hatchery would have more feather pecking, more feather damages, and lower reduced weight and egg production compared to chicks incubated, hatched and handled under calm circumstances.

## Material and methods

### Ethical note

All experimental protocols were approved by Linköping Council for Ethical Licensing of Animal Experiments, ethical permit no 14916–2018 (Linköping, Sweden). Experiments were conducted in accordance with the approved guidelines.

### Animals and housing

All animals used in this study were White Leghorn chicks from the Lohmann LSL strain (Lohman Tierzucht, Germany). Two groups of animals were used–experimental and control chicks–and these were from the same parental flock, and from eggs collected during the same time period.

100 female experimental chicks (hatchery chicks, HC) were obtained from the commercial hatchery Gimranäs AB, Herrljunga, Sweden. These chicks were processed according to commercial standard routines as following: At incubation day 22, i.e., one day after average hatching, the racks with chicks were taken out of the incubator and tilted on a belt. After the shells were removed by hand, the chicks were conveyed to a sex sorting station where they were manually sorted by wing inspection. The males were culled according to commercial routines, and females were further conveyed to a second station where they were manually vaccinated against Marek’s disease using automatic dispensing machines. Once vaccinated, the animals were moved to a high-speed conveyer belt system with multiple drops to spatially separate them before machine counting. Chicks were then automatically machine counted, packed in boxes and transported for 3.5 hours to Linköping University. On arrival, they were placed in rearing pens and from this point on, treated the same as control chicks.

As controls (control chicks, CC), 200 fertilized eggs were placed in a small incubator at Linköping University, and the hatchlings were removed from the incubator at the same time point as HC. CC were carefully handled, and sex sorted by wing inspection, the males were culled, and the females (n = 88) were immediately placed in rearing pens. CC were wing tagged and vaccinated for Marek’s disease at three weeks of age. At this same time point, HC were also wing tagged and sham vaccinated in order to treat the controls as similar as possible to HC after arrival at the hatchery. The reason why HC and not CC were vaccinated at hatch was that vaccination is a part of the commercial hatchery treatment which CC were not exposed to. The vaccination procedure, as well as the transportation, are likely to affect the newly hatched chicks, and the aim of the study was to assess the complete procedure during the first day of the hatchery chicks’ life. To consider possible weight loss or weight gain post-hatch, control chicks were not weighed immediately after hatch but at the time when the hatchery chicks arrived to the experimental facility, and the two groups were provided with feed at the same time.

Throughout the whole experiment up to 25 weeks of age, HC and CC were kept in the same rooms but in separate pens. After hatching, they were evenly distributed in four identical 0.9x0.9 m pens. These pens were increased in size to 0.9x2.7 m when the chicks were two weeks old and stayed this size up to five weeks of age when the animals were moved to the adult chicken facility. From five to 25 weeks of age, the birds were held in two identical multilevel pens measuring 2.6 x 3 x 3 m. They were kept on sawdust and were provided with ad lib food and water, perches, and from five weeks of age, nest boxes.

### Recordings

All chicks were weighed at hatch (n_CC_ = 72, n_HC_ = 95) and thereafter at 4 days (n_CC_ = 71, n_HC_ = 90), one week (n_CC_ = 77, n_HC_ = 97), two weeks (n_CC_ = 84, n_HC_ = 99), three weeks (n_CC_ = 87, n_HC_ = 98), five weeks (n_CC_ = 87, n_HC_ = 99), eight weeks (n_CC_ = 88, n_HC_ = 100), 20 weeks (n_CC_ = 48, n_HC_ = 51) and 25 (n_CC_ = 46, n_HC_ = 51) weeks of age.

From 19–25 weeks of age, eggs from each pen were collected daily on each Tuesday-Thursday and egg weight and number of eggs per pen was recorded. Since a hen does not lay more than one egg per day, each day could be considered an independent replicate of the egg laying in that group. Standard error was estimated as the average standard error for each group on each of the days.

At 15 weeks of age, a feather pecking study was performed. Seven birds were gently carried from their home pen and placed in a square test arena measuring 3 m^2^. The arena consisted of an evenly lit empty space covered with saw dust. No food or water was provided in the pen. After an acclimatisation period of 30 minutes, the birds were continuously observed for 30 minutes and every peck at head, tail and body was recorded. Each feather peck was scored as either “gentle” (gentle winkle another bird’s feathers), “severe” (harshly peck and pluck one or more feathers from another bird). In addition, “peck” was recorded for aggressive pecks (harshly peck at another bird without pulling feathers). In total, 12 replicates with seven birds each from each treatment were tested.

At 25 weeks of age, a total of 100 chicks (n_CC_ = 49, n_HC_ = 51) were scored for feather damages at head, back, belly, wings and tail, and in addition for damage and wounds on combs and wattles. For the feather damage scoring, the following scale was used: 0: No damage; 1: A few feathers ruffled with little damages; 2: Many (>5) feathers ruffled with some damages, or missing feathers; 3: Many (>5) feathers heavily ruffled and with major damage to more than one feather, or loss of feathers. The scores for the different body parts were added to obtain a total feather score.

For comb and wattle, the following scale was used: 0: No damage; 1: Some (1–2) small bruises; 2: Many (≥3) bruises of small to severe character; 3: Heavily damaged with many severe bruises and open wounds. The same person, who was blind for treatment, performed all feather and comb-wattle scoring.

### Statistics

An independent t-test was used to evaluate any significant weight differences between the groups. The egg data were pooled into weeks. Number of eggs per day and pen was used as the independent replicate in this comparison. These data were then analyzed with a generalized linear model with the scale response “linear” and the link function “identity”, with treatment and age as factors. For the data obtained in the feather pecking behaviour test, the scores from “severe FP” and “pecks” were pooled, whereas “gentle FP” was regarded as a different behaviour and therefore analyzed separately. Both were analyzed with independent sample t-tests. Assessment of differences in feather condition was made with Mann-Whitney U test. All statistical analyses were carried out in SPSS. Differences were considered significant when P<0.05, and we considered a tendency to be present when P<0.1.

## Results

**Fig 1:**
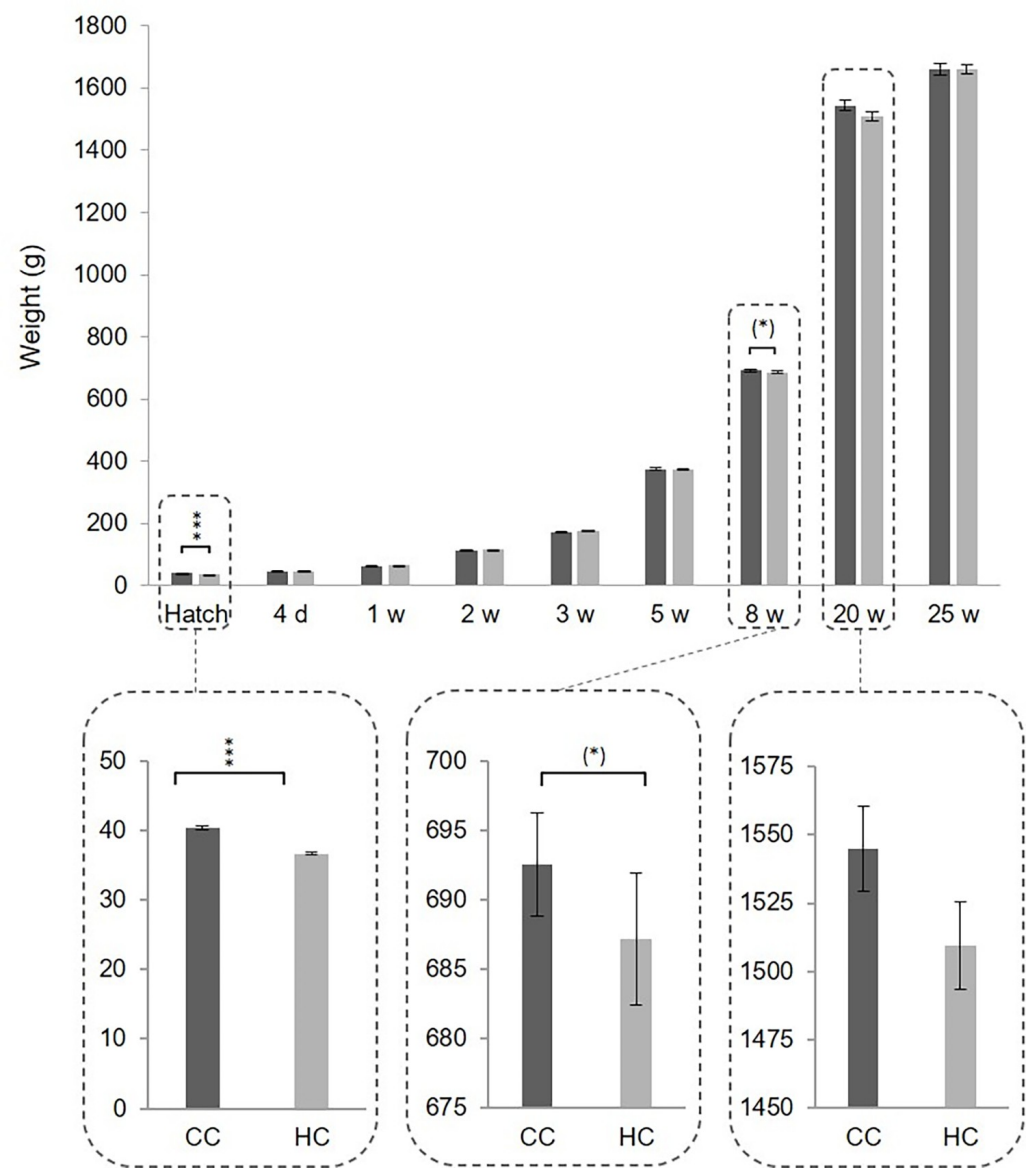
Weight at different ages in chicks from a commercial hatchery (HC) and control chicks (CC), (*)p<0.1, ***p<0.001. At 20w of age, p = 0.12.

There was a difference in hatch weight between the two groups where HC weighed significantly less than CC (Fig 1, t(165) = 8.610, p<0.001), but there were no significant differences at any other age. However, there was a tendency that CC weighed more than HC at 8 weeks of age, shortly after having been moved to a new facility (Fig 1, t(186) = 1.647, p = 0.10) and at 20 w of age (Fig 1, t(96) = 1.569, p = 0.12).

**Fig 2:**
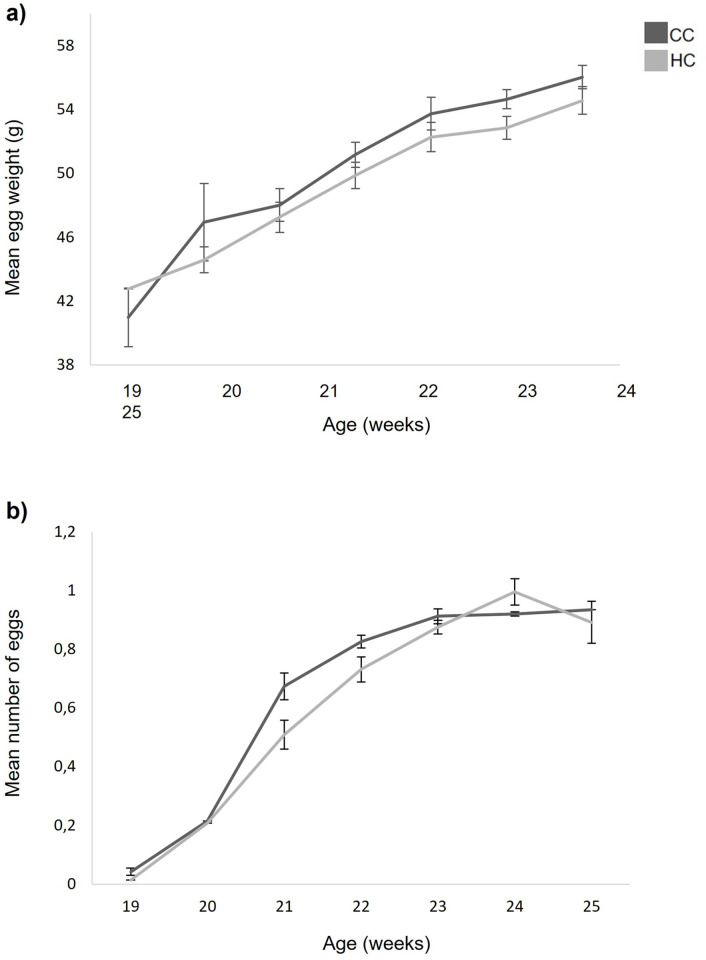
Egg production in hens from a commercial hatchery (HC) and control hens (CC), a) mean number of eggs (±SEM) per chick per day for each week, and b) mean weight (±SEM) of all eggs laid during each week.

With respect to egg production, both numbers of eggs and average egg weight significantly increased with age (Fig 2A χ^2^ = 1893.425, df = 6, p<0.001; Fig 2B, χ^2^ = 428.404, df = 6, p<0.001). In both periods, there was a significant difference between the two treatment groups where CC produced more (Fig 2A, χ^2^ = 4.262, df = 6, p<0.05) and heavier eggs (Fig 2B, χ^2^ = 6.332, df = 6, p<0.05) than HC.

**Fig 3:**
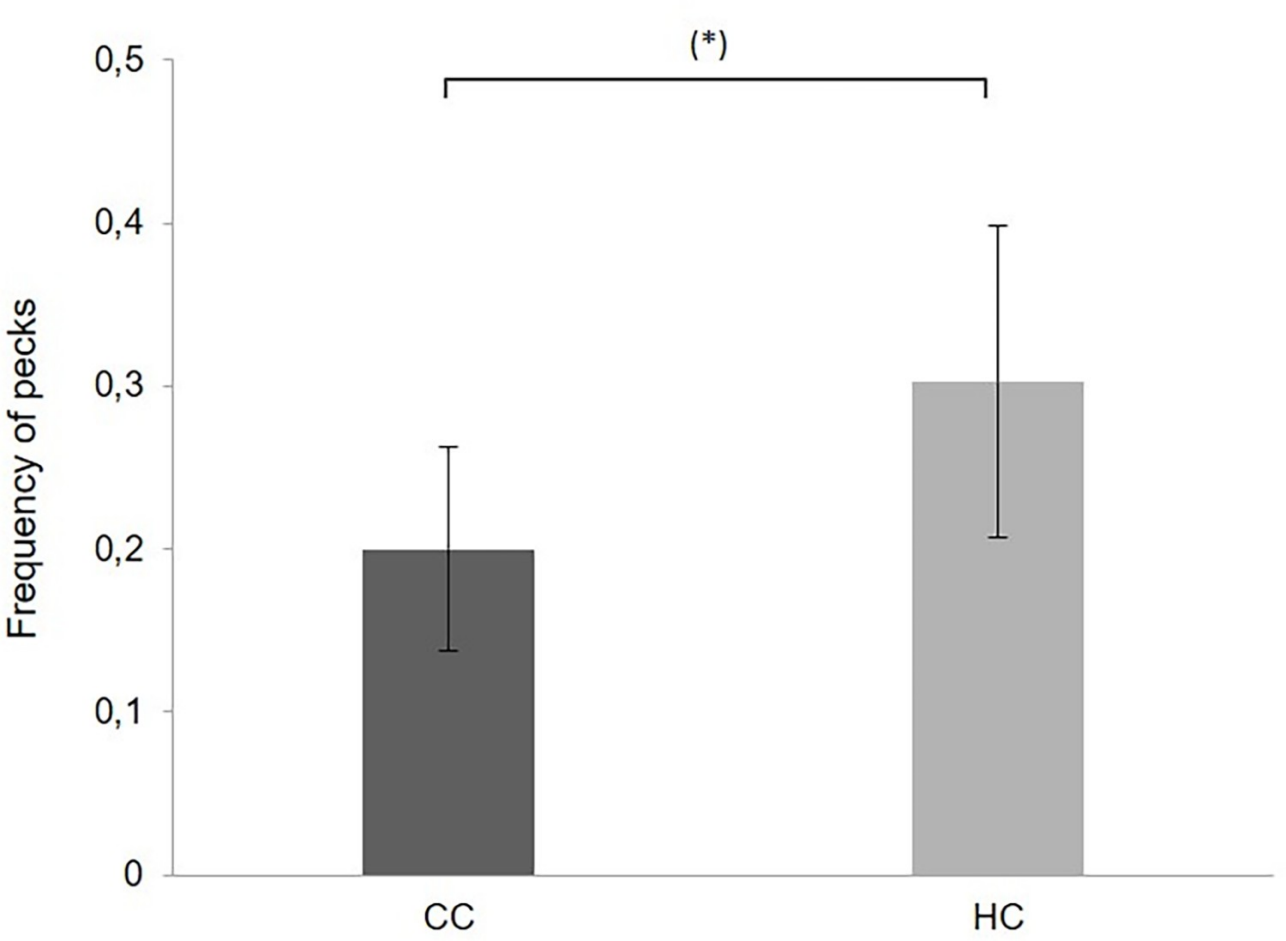
Severe feather pecking and pecks (nrs of pecks per minute per group (±SEM), in chicks from a commercial hatchery (HC) and control chicks (CC) at 15 w of age, (*) p<0.1.

During the test at 15 weeks of age, there was a tendency for HC to perform more severe feather pecking and pecks than CC (Fig 3, t(22) = -0.858, p = 0.093. There was no significant difference in gentle feather pecking (t(22) = -1.109, p = 0.279).

**Fig 4:**
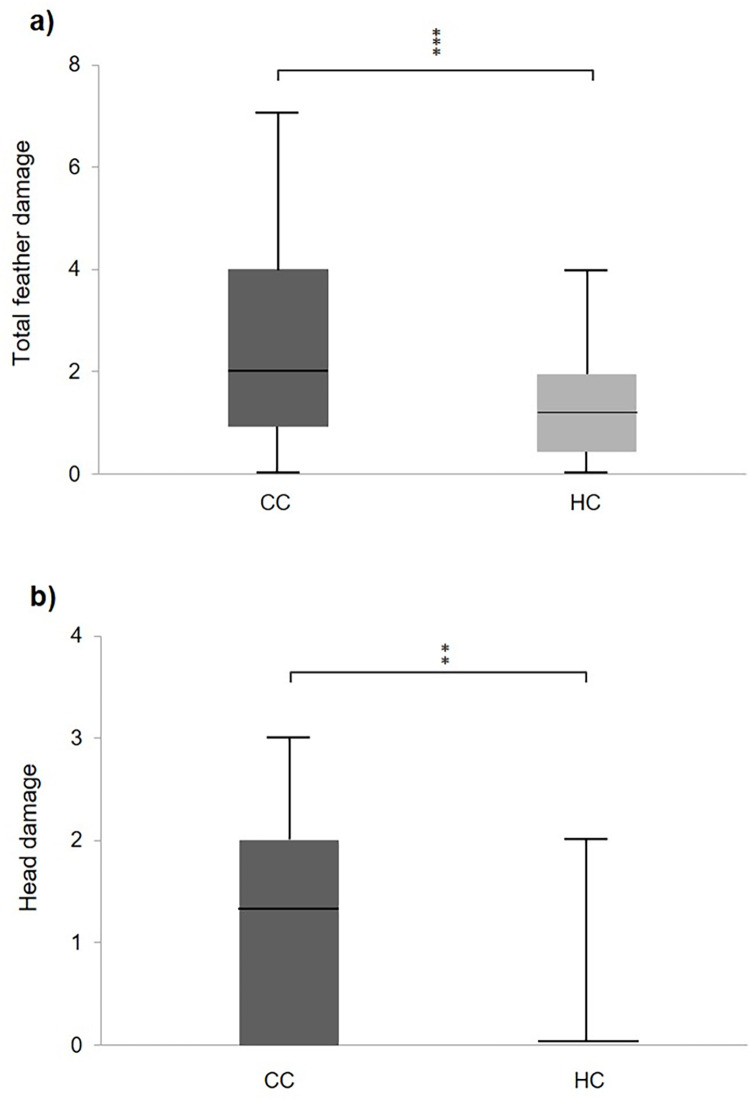
**a, b** Box plots showing the median scores for feather damages on chicks from a commercial hatchery (HC) and control chicks (CC) at 25 w of age where, in Fig 4A) total feather damage, the scale runs from 0–15, and in Fig 4B) from 0–3. The box includes +/- upper and lower quartile and the range includes max and min values. **p<0.01), ***p<0.001.

At 25 weeks of age, CC had significantly more total feather damages than HC (Fig 4A, U = 712.500, p<0.001). When analysing the body parts separately, there was a significant difference between the treatments in feather damages at head (Fig 4B, U = 622.000, p<0.01) and a tendency for belly (U = 1117.500, p = 0.093). There were no differences between the treatments with respect to feather damages on back (U = 1208.500, p = 0.671), wings (U = 1239.500, p = 0.938) or tail (U = 1150.500, p = 0.434). There were no significant differences between treatments with respect to wounds and injuries on comb (U = 1079.500, p = 0.126) or wattle (U = 1247.500, p = 0.977).

## Discussion

In this study, it was shown that chickens that were exposed to incubation, hatching and handling in a commercial hatchery weighed less after hatch and tended to do so also at eight weeks of age following a transport and change of environment. Chicks from the commercial hatchery also tended to perform more feather-pecking (FP) behaviour at 15 w of age and laid fewer and smaller eggs. However, control chickens incubated, hatched and handled under calm circumstances had more feather damages at 25 w of age while there was no difference in injuries at comb and wattle.

After hatch and transport, HC weighed significantly less than CC. It was previously shown that chicks from a commercial hatchery have an elevated level of CORT at hatch as well as after the hatchery procedure [3]. The HPA-axis starts to develop early in the chicken embryo where adrenocorticotropic hormone can be detected at day 7 of incubation [30]. Previous research has shown that stressors pre-hatch, such as loud noise [31], reduced gas exchange [15] and increased CORT levels [16], can affect weight and growth in chickens. The stress that was imposed by incubation, hatching and processing in the commercial hatchery followed by transport therefore affected the chicks’ weight negatively. In a previous study, no differences could be detected in down-feather CORT-levels between newly hatched chicks from commercial or control conditions [4]. Such levels reflect the stress experienced in-ovo during incubation. Together with the present results, this indicates that the major part of the stress affecting the chicks occur after hatch, corroborating other earlier studies [2].

Even though early life stress impacts chicks’ weight and growth, they seem to catch up by compensatory growth in just a few weeks [9, 11, 14]. Hence, the difference in weight between stressed and non-stressed chicks is generally not long lasting, which can explain why there was no differences in weight later in life between HC and CC. However, there was a tendency for the weight to differ between the treatments at eight weeks of age. An explanation for this can be that the chickens at five weeks of age were moved to a new facility and placed in an entirely new environment. A previous experiment has shown that the HCs HPA-axis is more sensitive than the CCs, and reacts stronger to stressors, even after five weeks [3]. Therefore, it is possible that the stress caused by moving to a new facility affected HC more than CC.

There was a difference in egg production where CC laid heavier and more eggs than HC. In a previous study, the opposite effect was found [3] and there can be several explanations for this. The egg production in the previous study was recorded during the first three weeks from onset of egg laying, which was around 15 w of age. Here, the egg collection started at week 19 and continued for six weeks, which is a more relevant time-period for the industry, since all birds are expected to have reached sexual maturity at this age. It might be that HC have an earlier onset of egg laying, but CC birds catch up after a couple of weeks. Another possible explanation for this difference could be a difference in quality and age of the parental birds, which was not controlled for in either of the studies (although both hatchery and control eggs within each study came from the same parental flock and were collected during the same time period). It is well known that the environment and the age of the mother hen affects the chicks in several ways due to epigenetic effects [32] as well as due to hormones such as CORT transferred to the egg [33]. The present results strongly suggest that the early stress associated with the hatching environment may affect later egg production, and it is quite likely that the effect is negative at least under some conditions.

When the birds were 15 weeks old, there was tendency for more FP behaviour among the HC. This is generally considered one of the major welfare problems in laying hens and is the reason for the widely criticized practice of beak trimming [34]. The results suggest that one way of reducing this behaviour could be to reduce the perinatal stress associated with commercial hatching.

When FP occurs in flocks of laying hens, this will negatively affect their feather condition. A study of Glatz [19] showed that egg production is correlated with feather damage, where birds with better feather cover produce more eggs. Somewhat unexpected, although HC showed more FP behaviour at 15 weeks of age, CC had more feather damages at 25 weeks of age. However, the main feather damages observed were at the head (and partly on the belly), whereas FP behaviour is usually directed towards the tail feathers and the lower part of the back [35]. This indicates that the differences observe in feather condition were not caused by FP. Other possible causes could be mechanical abrasion due to uncontrolled differences in the pen furniture. The fact that there were no differences in comb and wattle damage indicates that there were no differences in aggressive behaviour between the groups which could explain the feather damage on the head. It should also be noted that most FP related damages in laying hens are seen after 25 weeks of age [36], and thus the feather scoring in the present study could have been carried out at a too young age to actually observe differences in feather condition.

In conclusion, this study shows that the stressful incubation, hatching and handling of chickens in a commercial hatchery was related to a reduced post-hatch weight, and a tendency for reduced weight gain. Furthermore, commercially hatched chicks had a reduced egg production and a tendency for increased feather pecking behaviour. A poorer feather condition in control chickens could possibly be the result of environmental impact rather than related to hatchery effects. The results suggest several long-time effects of commercial hatching routines that could be negative for animal welfare as well as for egg production.

## Supporting information

S1 Data (XLSX)
